# Supplementary material for: CNOT6 deadenylase safeguards postnatal growth and metabolic transition via Fgf21 mRNA decay
Source: bioRxiv. 2026 Feb 18:2026.02.17.706459. Preprint. [Version 1] doi: 10.64898/2026.02.17.706459 (PMC12934697; doi:10.64898/2026.02.17.706459)

bioRxiv preprint doi: https://doi.org/10.64898/2026.02.17.706459; this version posted February 18, 2026. The copyright holder for this preprint (which was not certified by peer review) is the author/funder, who has granted bioRxiv a license to display the preprint in perpetuity. It is made available under aCC-BY 4.0 International license.

Supplemental Information

Figure S1. *Cnot6l* deletion does not affect organ growth during postnatal development

(A-L) Weights and representative images of tissues, including livers (A-B), kidneys (C-D), spleens (E-F), hearts (G-H), lungs (I-J), and brains (K-L) of *Cnot6l* WT and KO male and female mice. n = 5-10 per group. All data are presented as mean ± SEM. Related to Figure 1.

Figure S2. Generation and validation of *Cnot6* KO mice and increased perinatal lethality

(A) Schematic representation of the *Cnot6* WT allele, targeting vector, and mutant allele. White triangles indicate loxP sites. Blue boxes represent reporter and selection cassettes (lacZ, β-galactosidase; neo, neomycin). Numbered black boxes indicate *Cnot6* exons. The targeting vector was designed to disrupt normal transcription through a gene-trap strategy, resulting in complete loss of detectable CNOT6 protein. (B) Genotyping PCR results for *Cnot6* WT, HET, and KO mice. (C) Western blot analysis confirming loss of CNOT6 protein expression in *Cnot6* KO mice. β-actin was used as a loading control. (D) Genotype distribution of live pups from *Cnot6* heterozygous intercrosses at day 0 (birth) and day 14, showing reduced survival of homozygous KO mice. Related to Figure 2.

Figure S3. *Cnot6* deletion impairs organ growth during postnatal development

(A-F) Weights and representative images of tissues, including lungs (A-C) and brains (D-F) of *Cnot6* WT and KO male and female mice. n = 5-38 per group. Red reference line indicates 10 mm. All data are presented as mean ± SEM. *$p < 0.05$, **$p < 0.01$, ***$p < 0.001$, ****$p < 0.0001$; p values by the Student's t test. Related to Figure 3.

bioRxiv preprint doi: https://doi.org/10.64898/2026.02.17.706459; this version posted February 18, 2026. The copyright holder for this preprint (which was not certified by peer review) is the author/funder, who has granted bioRxiv a license to display the preprint in perpetuity. It is made available under aCC-BY 4.0 International license.

Figure S4. RNA-seq analysis of *Cnot6* WT and KO livers

(A) Principal Component Analysis (PCA) of male Cnot6 WT and KO liver RNA-seq datasets. Each point represents one biological replicate. PCA was performed using the plotPCA function in R based on log_2-transformed normalized gene counts. WT and KO samples form distinct clusters, indicating clear genotype-dependent transcriptional profiles.

Table S1. Differentially expressed genes in livers of 2-week-old *Cnot6* WT and KO mice

Differential expression analysis was performed using DESeq2. Genes with $|log_2$ fold change$| \geq 1$ and adjusted $p \leq 0.05$ were considered significant (n = 378).

Table S2. Primer sequences used for qPCR

Forward and reverse primers used in this study.

Table S3. Gene-level RNA-seq read counts from Cnot6 WT and KO mouse livers

Gene-level raw read counts obtained from bulk RNA-seq analysis of liver tissues from 2-week-old *Cnot6* WT (WT1-WT3) and KO (KO1-KO3) mice. Counts represent unnormalized read numbers prior to differential expression analysis.

bioRxiv preprint doi: https://doi.org/10.64898/2026.02.17.706459; this version posted February 18, 2026. The copyright holder for this preprint (which was not certified by peer review) is the author/funder, who has granted bioRxiv a license to display the preprint in perpetuity. It is made available under aCC-BY 4.0 International license.

Figure S1

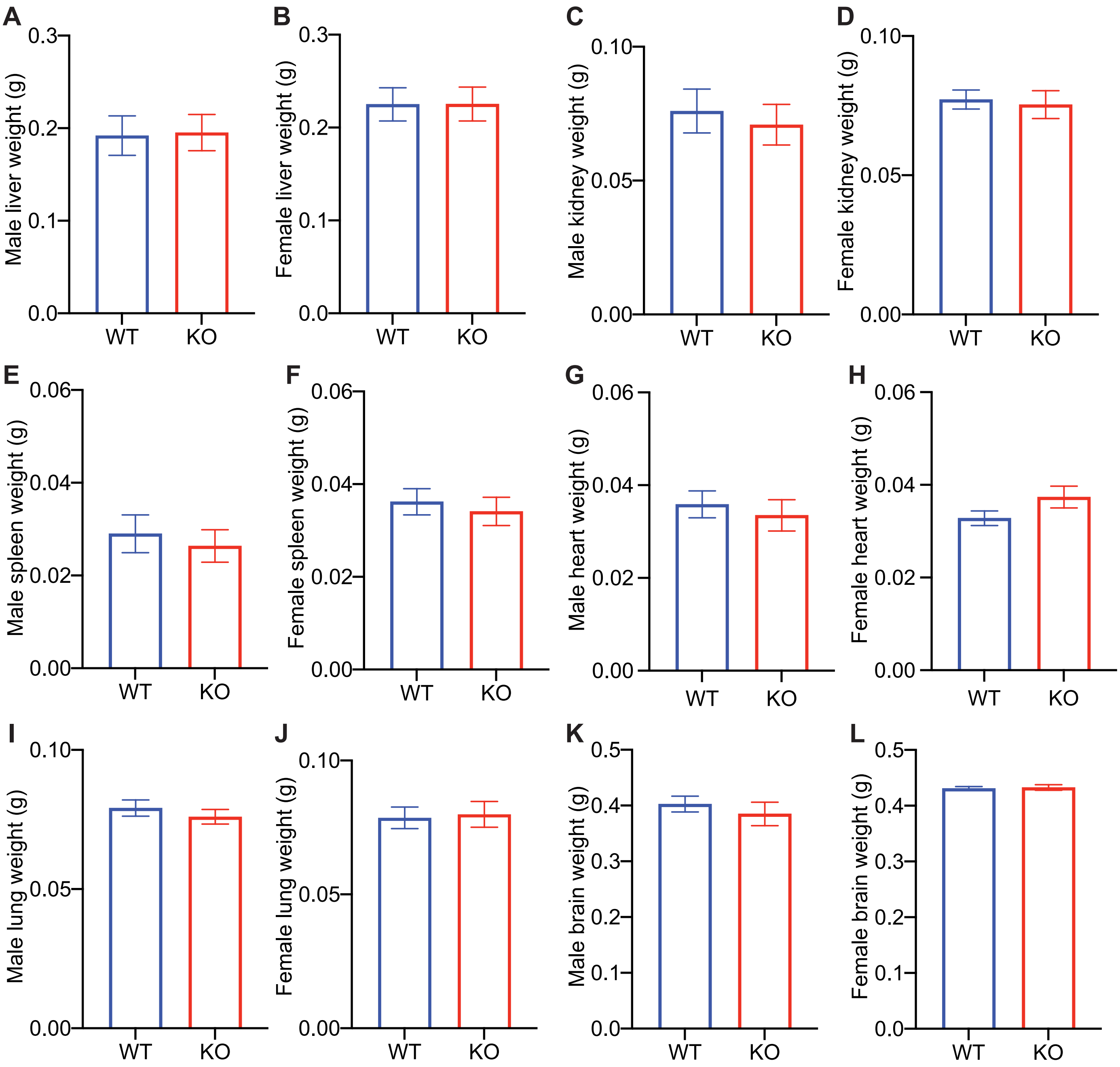

bioRxiv preprint doi: https://doi.org/10.64898/2026.02.17.706459; this version posted February 18, 2026. The copyright holder for this preprint (which was not certified by peer review) is the author/funder, who has granted bioRxiv a license to display the preprint in perpetuity. It is made available under aCC-BY 4.0 International license.

Figure S2

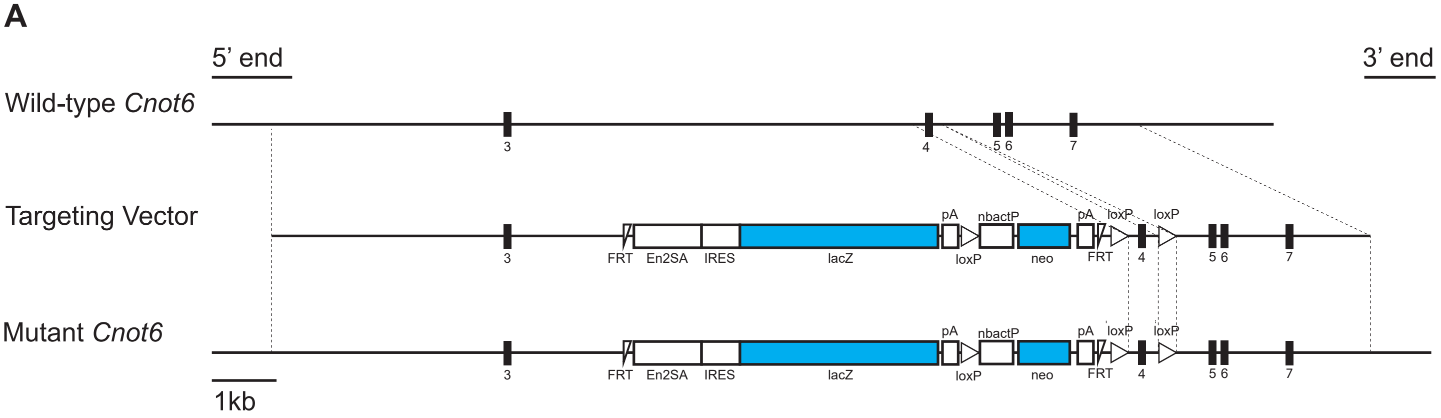

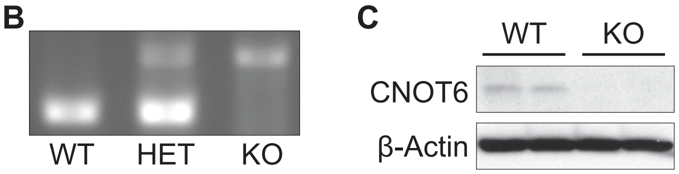

D

	WT	HET	KO
Day 0	133	153	72
Day 14	113	125	37

bioRxiv preprint doi: https://doi.org/10.64898/2026.02.17.706459; this version posted February 18, 2026. The copyright holder for this preprint (which was not certified by peer review) is the author/funder, who has granted bioRxiv a license to display the preprint in perpetuity. It is made available under aCC-BY 4.0 International license.

Figure S3

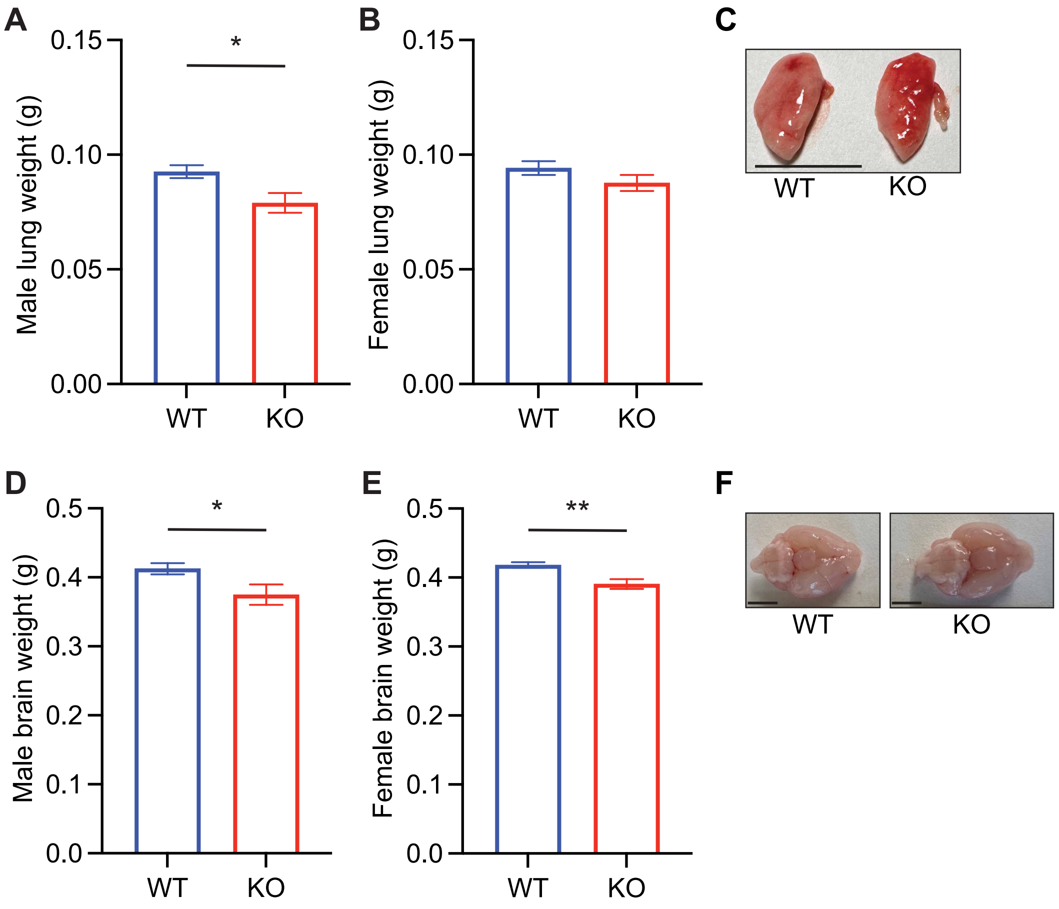

bioRxiv preprint doi: https://doi.org/10.64898/2026.02.17.706459; this version posted February 18, 2026. The copyright holder for this preprint (which was not certified by peer review) is the author/funder, who has granted bioRxiv a license to display the preprint in perpetuity. It is made available under aCC-BY 4.0 International license.

Figure S4

A

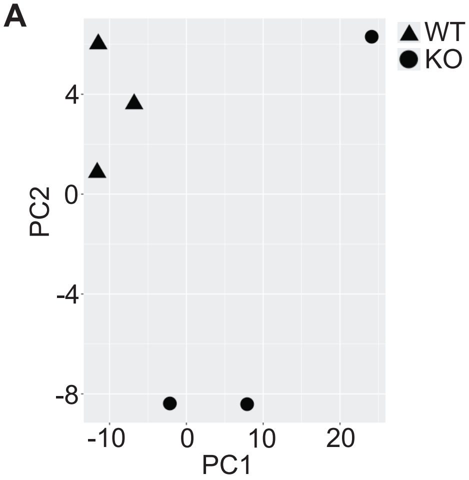

Supplement: Supplement 4 [file NIHPP2026.02.17.706459v1-supplement-4.pdf]
